# Microscopic Evaluation, Molecular Identification, Antifungal Susceptibility, and Clinical Outcomes in *Fusarium*, *Aspergillus* and, Dematiaceous Keratitis

**DOI:** 10.1155/2013/605308

**Published:** 2013-10-24

**Authors:** Devarshi U. Gajjar, Anuradha K. Pal, Bharat K. Ghodadra, Abhay R. Vasavada

**Affiliations:** ^1^Department of Microbiology and Biotechnology Centre, Faculty of Science, M. S. University of Baroda, Vadodara 390 002, India; ^2^Iladevi Cataract and IOL Research Centre, Ahmedabad, Gujarat 380052, India

## Abstract

*Purpose*. *Fusarium*, *Aspergillus*, and Dematiaceous are the most common fungal species causing keratitis in tropical countries. Herein we report a prospective study on fungal keratitis caused by these three fungal species. *Methodology*. A prospective investigation was undertaken to evaluate eyes with presumed fungal keratitis. All the fungal isolates (*n* = 73) obtained from keratitis infections were identified using morphological and microscopic characters. Molecular identification using sequencing of the ITS region and antifungal susceptibility tests using microdilution method were done. The final clinical outcome was evaluated in terms of the time taken for resolution of keratitis and the final visual outcome. The results were analyzed after segregating the cases into three groups, namely, *Fusarium*, *Aspergillus*, and Dematiaceous keratitis. *Results*. Diagnosis of fungal keratitis was established in 73 (35.9%) cases out of 208 cases. The spectra of fungi isolated were *Fusarium* spp. (26.6%), *Aspergillus* spp. (21.6%), and Dematiaceous fungi (11.6%). The sequence of the ITS region could identify the *Fusarium* and *Aspergillus* species at the species complex level, and the Dematiaceous isolates were accurately identified. Using antifungal agents such as fluconazole, natamycin, amphotericin B, and itraconazole, the minimum inhibitory concentrations (MICs) for *Fusarium* spp. were >32 **μ**g/mL, 4–8 **μ**g/mL, 0.5–1 **μ**g/mL, and >32 **μ**g/mL, respectively. Antifungal susceptibility data showed that *Curvularia* spp. was highly resistant to all the antifungal agents. Overall, natamycin and amphotericin B were found to be the most effective antifungal agents. The comparative clinical outcomes in all cases showed that the healing response in terms of visual acuity of the Dematiaceous group was significantly good when compared with the *Fusarium* and *Aspergillus* groups (*P* < 0.05). The time required for healing in the *Fusarium* group was statistically significantly less when compared with the *Aspergillus* and Dematiaceous groups. *Conclusion*. This study demonstrates important differences in microscopic features of scraping material and antifungal susceptibility between the three groups. Early and accurate identification coupled with the MIC data, and thereby appropriate treatment is crucial for complete recovery.

## 1. Introduction

Mycotic keratitis is an important ophthalmic problem causing visual disability due to its protracted course and unfavorable responses. The incidence of fungal keratitis has been reported to range between 25.6% and 36.7% in various parts of India [1–4]. It is evident that *Aspergillus *and *Fusarium* are the most common species causing keratitis in tropical countries including India, whereas pigmented Dematiaceous fungi are the third most common cause of mycotic keratitis [1, 5–7]. Studies on their molecular identification, antifungal susceptibility, and comparisons with the clinical outcomes would be of great importance, as the pathogenic potential may vary between these genera. The most widely sequenced DNA region in fungi is the ITS region, and the International Sub-Commission of fungal bar coding has proposed the ITS region as the prime fungal bar code for species identification [8]. Molecular identification of keratitis causing *Fusarium* and *Aspergillus* isolates has been reported earlier [9–12]. Fungal ulcers are commonly treated empirically; drugs are typically selected without regard to susceptibility data. This is because the antifungal susceptibility testing takes time and needs trained personnel to perform the testing. There are four studies that report the *in vitro* antifungal susceptibility patterns among keratitis-causing fungal isolates from India [13–16]. On the other hand nonophthalmic Fusaria have been reported to exhibit greater resistance to antifungal agents continuously over a period of time [17, 18]. Hence, periodic reports from different geographical areas would help record the variations over a period of time and at the same time help in modulating the current treatment options. We report a prospective study to compare different aspects of fungal keratitis such as its clinical features, microbial evaluation, molecular identification, antifungal susceptibility, and clinical outcomes.

## 2. Materials and Methods

Patients were recruited after an informed consent was obtained from all subjects. The study followed the declaration of tenets of Helsinki and was approved by the Institutional Ethical Review Board.

### 2.1. Clinical Examination

A prospective study of patients with keratitis was conducted during the period from June 2009 to May 2012. All the patients were examined with a standard written protocol that included a detailed history with regard to the duration of symptoms, predisposing factors, the exact nature of trauma, immediate treatment administered contact lens usage, previous history of ocular surgeries, history of diabetes, and usage of topical or systemic steroids. The same consultant doctor, as per the standard protocol approved by the institutional review board, performed a thorough examination of the involved and fellow eyes. The same consultant ophthalmologist filled a form. In this form, the following aspects were documented: the presence or absence and form of the following clinical features, elevation of slough (raised or flat), texture of slough (wet or dry), ulcer margins (serrated or well defined), size of the abscess, pigmentation, Descemet's folds, satellite lesions, dendritic lesions, immune ring, hypopyon, fibrin, flare or cells in the anterior chamber, deep lesions, and endothelial plaque. Clinical photographs were taken using the Haag Straight slit lamp microscope with a photo slit attachment.

### 2.2. Clinical Specimens and Microbiological Investigations

Corneal scrapings were taken from patients when at least one of the following was present: size of the infiltration was >2 mm with an epithelial defect, depth of the infiltrate was >20% of the corneal thickness, the anterior chamber reaction was > grade 2, evidence of any organic trauma, or failure to regress in 24 hours. Local anesthetic eye drops (proparacaine 0.5%) were instilled to the affected eye to minimize ocular discomfort and facilitate the corneal scraping procedure. Scrapings were obtained aseptically from the base and edges of each ulcer using a disposable blade. A part of the scraping material was examined for the presence of fungi, bacteria, or acanthamoeba by using 10% KOH–0.05% calcofluor white stain wet mounts and Gram staining [19]. The scraping material was also directly inoculated in blood agar, Sabouraud's dextrose agar (SDA), and chocolate agar media (Himedia, Himedia Pvt Ltd, Mumbai) which were incubated at 37°C and 28°C and in 5% CO_2_, respectively. A diagnosis of fungal keratitis was made when at least one of the following was confirmed: a corneal scraping examination revealed fungal hyphae in wet mounts or smears; the same fungus grew in the two culture media used; or the fungus grew confluently at all the inoculated sites on a single media. Microscopic pictures of the KOH wet mounts for all samples were taken under the light microscope and fluorescence microscope. The average width of 25 randomly selected hyphae, the distance between the septa, and the diameter of the chlamydospore-like structures whenever present were measured from digitalized photographs at 400x magnification using Biovis Image Plus Software v.4.11 (Expert Vision, Mumbai, India). Pictures of media plates showing fungal growth were taken at 24-hour growth, 48-hour growth, and 72-hour growth. Colour, diameter, and presence or absence of spores were observed for all fungal colonies on SDA and blood agar plates.

### 2.3. Morphological and Molecular Identification

Pure cultures of all isolates were maintained on Potato Dextrose agar (PDA). Cultures were examined using the lactophenol blue mount for sporulation at the end of 10, 20, and 30 days. The morphological and microscopic identification was done by growth characteristics, and microscopic characteristics, respectively. All morphological and microscopic characteristics were confirmed by comparing them with the characters given in the “Atlas of clinical fungi” [20]. Cultures that failed to sporulate on SDA and PDA were subcultured on oatmeal agar and carnation leaf agar. For molecular identification of the fungus, sequencing of the ITS (internal transcribed spacer) region was done. The DNA was extracted from the pure culture of the fungus grown on SDA using Zymo Research DNA isolation kit. After extraction, the DNA was amplified using ITS 1 (F-5′-TCCGTAGGTGAACC-3′) and ITS 4 primers (R-5′TCCTCCGCTTATTGATATGA-3′), which amplify the following genes of the fungal genome: partial 18S rRNA gene, complete ITS1, 5.8S rRNA gene and ITS2 regions, and partial 28S rRNA gene. Annealing temperature was 55°C for 1 minute. The size of amplicon produced after PCR reaction was around 500–600 base pairs for all fungi used in the present study. Sequencing of the ITS region was done at First Base Laboratories Sdn. Bhd, Malaysia, using primers ITS1 and ITS4. Sequences were obtained using both forward and reverse primers. Chromatogram processing, quality control, and editing of the sequences were done using BioEdit Software. Both sequences were aligned, and a final sequence was created. This final sequence was used for the BLASTN similarity search (http://www.ncbi.nlm.nih.gov/BLAST) and was also submitted to NCBI. For identification, only complete ITS1-5.8S-ITS2 entries of reference isolates in the BLAST database were taken into consideration. Complete identification was considered when a percent sequence similarity of >98% with a BLAST search expected value of zero was obtained.

### 2.4. Antifungal Susceptibility Testing

*In vitro* antifungal susceptibility testing was done against natamycin (Natamet; 5% suspension; Sun Pharmaceuticals Ind. Ltd, Halol, India), itraconazole (Itral; 1% suspension; Jawa Pharmaceuticals, Gurgaon, India), fluconazole (Nufl ucon; 0.3% suspension; NuLife Pharmaceuticals, Pune, India), and amphotericin B (RM 462, Himedia Labs Ltd, Mumbai, India) using the microdilution method and following the Clinical and Laboratory Standards Institute (CLSI) guidelines [21]. All antifungal agents were dissolved in DMSO and fluconazole was dissolved in water. The inoculums were prepared by covering the 7-day-old culture plate with normal saline (0.85% NaCl). This was followed by gentle probing of the colonies with the help of a pipette and adjusting the densities of the suspension (read at 530 nm) to a final inoculum of 0.5 McFarland standard. The final drug concentration range prepared using serial dilution were 0.008 to 132 *μ*g/mL for all the four antifungal agents. All the antifungal agents were tested in RPMI 1640 media with 2% glucose and without sodium carbonate.

### 2.5. Treatment Regime and Evaluation of Clinical Outcomes

Subsequent to the microscopic examination, if a positive report of fungal filaments was received, antifungal topical therapy with 5% natamycin was started for all cases immediately. One-hourly topical eye drops were applied around the clock for the first three days followed by two-hourly drops during waking hours until resolution of the ulcers. Patients also received 1% atropine sulphate eye drops. Systemic fluconazole (150 mg once a day) was prescribed for all patients with corneal stromal infiltrate extending beyond one-third of the cornea. After treatment, an ulcer was considered to be healed when the epithelial defect was <1 mm in diameter with a visible scar under slit lamp biomicroscopy. A healing time of less than 3 weeks from presentation was considered a good result and healing time of more than three weeks was considered a poor response. The responses to treatment were categorized into three groups as follows: perception of light to the Snellen's chart (good response), no change in visual acuity after treatment (poor response), and slight change in the visual acuity (slight response). Initial and final visual acuity was comparedfollowingtreatment using statistical analysis. The time for complete healing was also compared among the isolates. The follow-up best-corrected visual acuity (BCVA) of patients treated with topical and oral antimicrobial agents was the visual acuity measured when the patient was cured (inactive corneal scar with intact epithelium). Visual acuities obtained using Snellen's chart were converted into logarithms of the minimum angle of resolution (logMAR) for data analysis.

### 2.6. Statistical Analysis

The test of proportion was used to evaluate the epidemiological features and risk factors. *In vitro* susceptibility results obtained from the three groups were statistically analyzed using the Kruskal-Wallis test. A post hoc pair wise comparison was also done. The Wilcoxon Signed Ranks Test was used to compare visual acuity before and after treatment in each group.

## 3. Results

### 3.1. Epidemiological Characteristics and Clinical Features

A total of 208 patients with keratitis were recruited during the period from May 2009 to June 2012. In all 73 patients who had culture-proven mycotic keratitis; the incidence of culture-proven mycotic keratitis was 35.0%. Out of these 73 patients, 26 (35.6%) were infected with *Fusarium* spp., 15 (20.5%) were infected with *Aspergillus* species, and 11 (15.0%) were infected with Dematiaceous fungi. The average age of the patients was 41.84 years in the *Fusarium* group, 52.46 years in the *Aspergillus* group, and 46.53 years in the Dematiaceous fungi group. There were more males than females in all three groups. The seasonal distribution showed that infection of all the three types of keratitis was highest in winter and attained statistical significance (Table 1). The risk factors such as trauma to the eye/ocular surgery were predominantly seen in the *Aspergillus* keratitis group whereas the incidence of entry of vegetative foreign bodies was mainly seen in the *Fusarium* group (Table 1). Table 1 shows the comparative evaluation of the clinical features in all the three groups. The area of infiltration was large (>4 mm) in the central visual axis in all the three groups. Further, the presence of satellite lesions, ring infiltrate, dry appearance, and stromal involvement was evident in most of the cases. The presence of hypopyon and pigmentation was mainly associated with the Dematiaceous group, and this attained statistical significance. The presence of endothelial plaque was mainly associated with *Fusarium* infections whereas dendritic lesions and Descemet's folds were mainly observed in the *Aspergillus* group.

### 3.2. Microscopic Evaluation and Growth Characteristics of the Scraping Material

**Figure 1:**
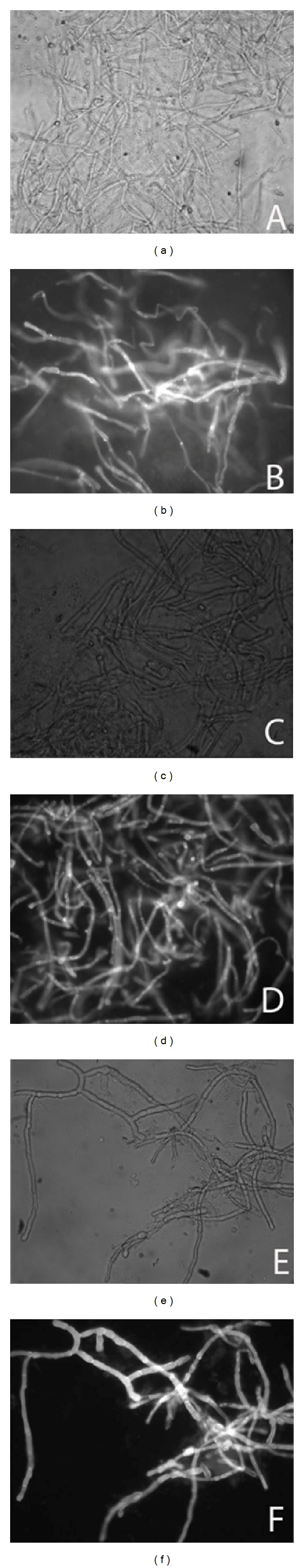
10% KOH mount of the scrapping material showing fungal hyphae, magnification ×400. Left panel: light microscopic picture, right panel: fluorescent microscopic picture taken after calcofluor white stain. (a and b) Scrapping material from *Fusarium* infections. (c and d) Scrapping material from *Aspergillus* infections. (e and f) Scrapping material from *Exserohilum* infections.

**Figure 2:**
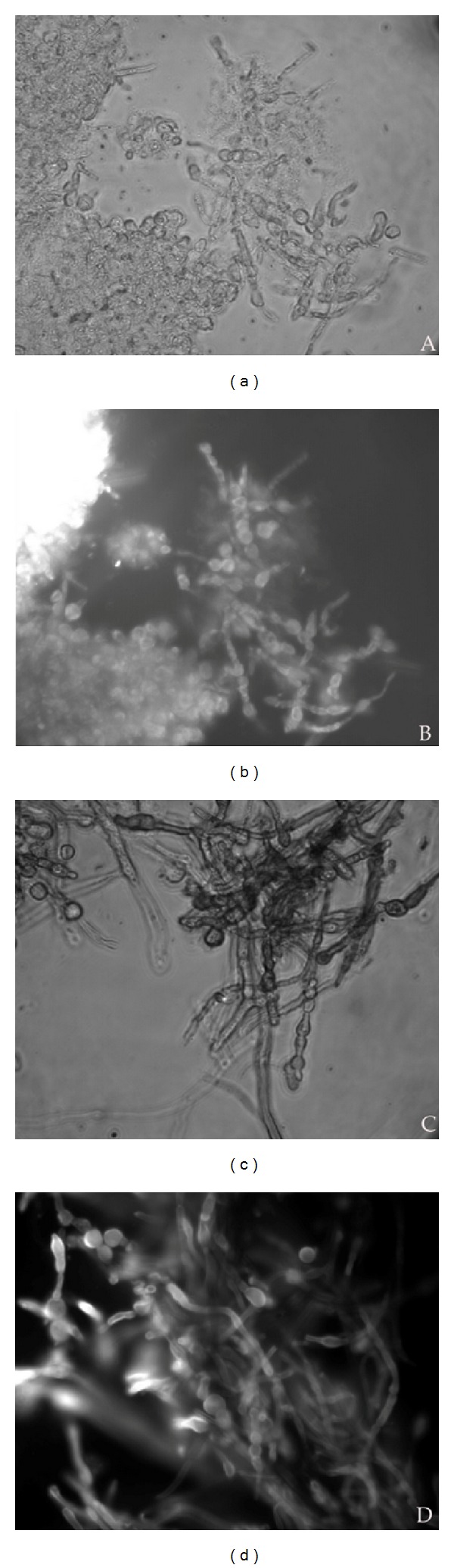
10% KOH mount of the scrapping material showing fungal hyphae, magnification ×400. Left panel: light microscopic picture, right panel: fluorescent microscopic picture taken after calcofluor white stain. (a and b) Scrapping material from *Fusarium delphinoides* infections. (c and d) Scrapping material from *Curvularia* infections.

**Figure 3:**
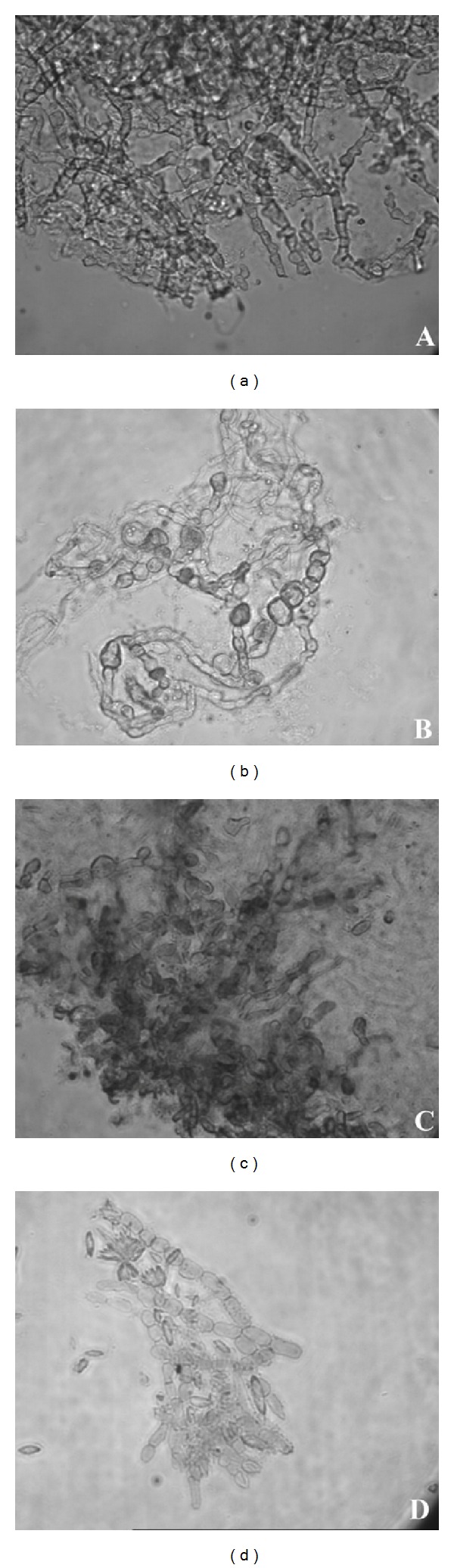
Light microscopic pictures of 10% KOH mount of the scrapping material showing fungal hyphae, magnification ×400. (a–d) Scrapping material from *Cladorrhinum*, *Curvularia*, *Papulaspora,* and *Cladosporium* infections, respectively.

All the samples of scraping material taken from the three groups showed the presence of large quantities of fungal filaments when seen under light and fluorescence microscopes (Figure 1). Samples from *Fusarium* infections (Figures 1(a) and 1(b)) showed the presence of fungal hyphae with an average thickness 3.87 ± 0.6 *μ*m. Septa were not visible under the light microscope (Figure 1(a)) but were clearly seen under the fluorescence microscope (Figure 1(b)). The distance between the septa was 21.65 ± 4.2 *μ*m. The average hyphal thickness in the *Aspergillus* group (Figures 1(c) and 1(d)) was 4.13 ± 0.65 *μ*m, and this was almost similar to the hyphal thickness in the *Fusarium* group. However, the distance between the septa was 12.84 ± 1.9 *μ*m. The average hyphal thickness in the Dematiaceous group was 8.79 ± 0.9 *μ*m (Figures 1(e) and 1(f)). Terminal and internal chlamydospore-like structures with a diameter of 9.44 ± 1.11 *μ*m were seen exclusively in all four samples from the *Fusarium delphinoides* group (Figures 2(a) and 2(b)). The scraping material from *Curvularia* infections also showed large quantities of chlamydospore-like structures with an average diameter of 11.15 ± 1.58 *μ*m (Figures 2(c) and 2(d)). These structures were absent in all the remaining samples of the Dematiaceous group. A huge variation in microscopic features was noticed in the Dematiaceous group (Figures 3(a)–3(d)). Scraping material, other than the *Curvularia* infection, showed a typical arrangement of septa.

**Figure 4:**
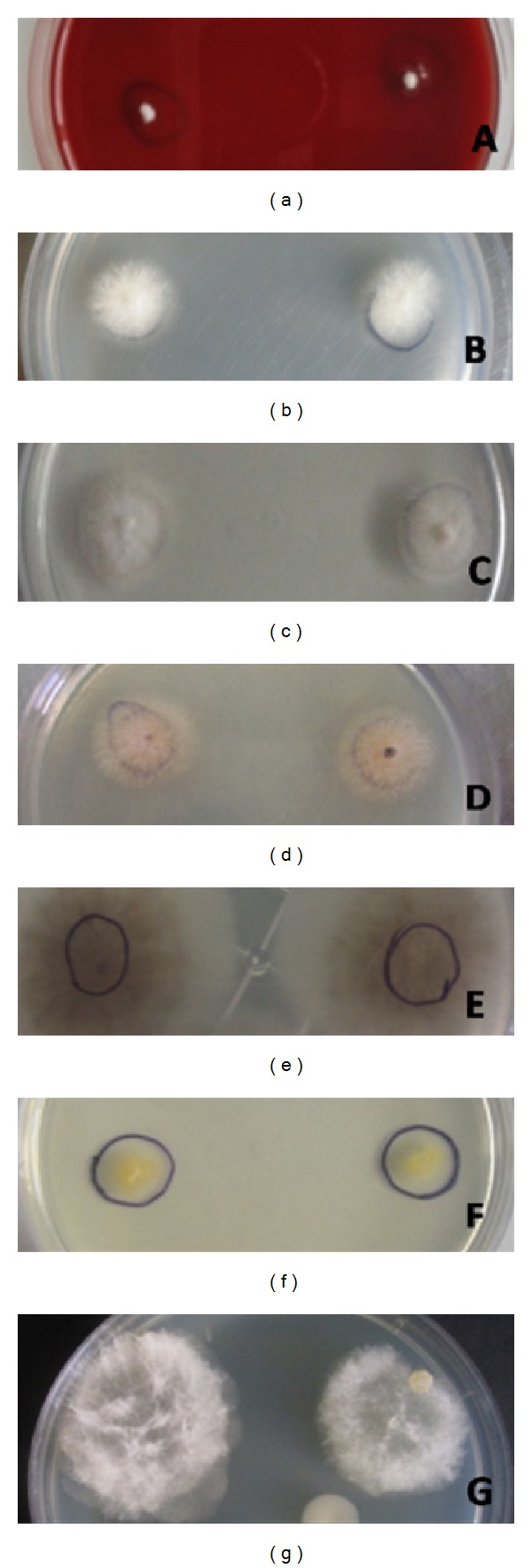
Growth of fungi from scrapping material after (a) 24 hrs, (b and c) *Fusarium* and *Aspergillus* samples after 48 hrs, (d and f) Dematiaceous samples, and (g) *Lasiodiplodia* sample.

A total of 47 (88.67%) out of 53 samples showed visible growth on all the media inoculated at 24 hours (Figure 4(a)). In the *Fusarium* group, 23 (88.4%) samples showed growth within 24 hours, while 3 samples showed growth within 48 hours. In the Aspergillus group, 13 (86.66%) samples showed growth within 24 hours and the remaining 2 samples showed growth within 48 and 72 hours, respectively. The growth of both *Fusarium* and *Aspergillus* samples on SDA at 48 hours was similar with respect to the growth rate (Figures 4(b) and 4(c)). In the Dematiaceous group, 11/12 (91.6%) samples showed growth on SDA within 24 hours. All the samples of the Dematiaceous group showed the presence of a peculiar color, for example, pink or light brown in case of *Curvularia* spp. (Figure 4(d)), yellow for *Papulaspora* spp. (Figure 4(e)), and dark brown for *Exserohilum* spp. (Figure 4(f)). A sample of *Lasiodiplodia theobromae* obtained from the scraping material was grown for 48 hours and a gray fluffy growth with abundant aerial mycelia was visible as seen in Figure 4(g). It was further observed that SDA did not support sporulation of *Curvularia* spp. and *Lasiodiplodia* spp. in all the samples.

### 3.3. Microscopic and Molecular Identification of *Fusarium*, *Aspergillus* and Dematiaceous Spp

All isolates in the *Fusarium* and *Aspergillus* groups were identified to the genus level by means of their morphological characteristics. The morphological evaluation of *Fusarium solani* appeared to be straight forward and this was further confirmed using the ITS sequences. However, when the sequences were evaluated using the *Fusarium* MLST website, the match was to the *Fusarium solani* species complex and not to *Fusarium solani* per se. Hence, all *Fusarium* isolates were named as members of the *Fusarium solani* species complex. All other isolates of *Fusarium* (*n* = 4) were identified as *Fusarium dimerum* using their morphological features. However, identification using the ITS sequences at NCBI BLAST was *F. delphinoides* isolates (*n* = 4), *Fusarium dimerum* (*n* = 1), and *Fusarium delphinoides* (*n* = 3) using the MLST database. In the *Aspergillus* group, *A. niger*, *A. flavus*, *A. terreus,* and *A. fumigatus* were identified using morphological features. *A. tamarii*, *A. tubingensis*, *A. versicolor*, and *A. sydowii* were only identified when the ITS sequences were available. In the Dematiaceous group, *Curvularia lunata* and *Exserohilum rostratum* were identified using their growth characteristics and typical spores, and this was confirmed using the ITS sequence. Using ITS sequences, all the other dematiaceous isolates were identified as *Lasiodiplodia theobromae, Cladorrhinum bulbilosum, *and* Cladosporium cladosporioides.* The ITS sequence misidentified only one isolate as *Chaetomium *spp. It was later identified as *Papulaspora* spp. on the basis of its typical microscopic features.

### 3.4. *In Vitro* Antifungal Susceptibility

Table 2 shows the results of antifungal susceptibility testing of all isolates. Antifungal results showed that amphotericin B and natamycin are the most effective antifungal agents against *Fusarium* spp. In the *Aspergillus* group, amphotericin B and itraconazole showed the lowest MIC against *A. flavus, A. terreus, A. tamarii, and A. tubingensis *whereas natamycin and amphotericin B showed good *in vitro activity* against *A. niger and A. sydowii.* In the Dematiaceous group, except *Curvularia*, all the other isolates were highly susceptible to the antifungal agents tested. Amphotericin B and natamycin showed good *in vitro* activity against *Curvularia lunata*.

### 3.5. Evaluation of Clinical Outcomes

Table 3 shows the comparative clinical outcomes in all cases. In the *Fusarium* group, 20 cases healed and 3 worsened, while 3 were lost to follow-up. The minimum time required for healing was 14 days, whereas the maximum time taken to heal in the case of one patient was 300 days. In the *Aspergillus *group, 12 out of 15 cases healed with topical and oral antifungal medical treatment and two cases required therapeutic keratoplasty, whereas one case caused by *A. tamarii* worsened. The minimum time required for healing was 30 days and the maximum time required was 330 days. In the Dematiaceous group, 11 cases healed and one was lost to follow-up. The minimum time taken to heal was 7 days while the maximum time was 300 days. The healing response in terms of visual acuity of the Dematiaceous group was significantly good when compared with the *Fusarium* and *Aspergillus* groups (*P* < 0.05). The time required for healing in the *Fusarium* group was statistically significantly less when compared with the *Aspergillus* and Dematiaceous groups.

## 4. Discussion

In the present study of 208 keratitis patients, fungal etiology was confirmed in 35% of the cases, where *Fusarium* spp. was the most common isolate followed by *Aspergillus* and Dematiaceous. This is comparable to most studies from India [1–5]. In India, *Aspergillus* is mainly reported as the most common isolate in the Northern region, [1, 2, 22, 23] while *Fusarium* is mainly reported in the Southern region [4, 24] and Dematiaceous fungi are reported to be the third most common fungi. We found two reports from Ahmedabad, *Fusarium* was reported in a study conducted in 2003–2005 [25] while *Aspergillus* was the most common isolate reported in a study conducted in 2007-2008 [26]. We have obtained a definite history of trauma with vegetative/agricultural bodies largely in patients with *Fusarium* keratitis, whereas trauma due to a factor other than vegetative material or any other ocular surgery was found to be largely associated with the *Aspergillus* group. Among the traumatic agents, plants and agricultural material like hay have contributed to 76%, 78.5%, and 61.2% of cases of keratitis in studies from Assam [27], Gujarat [25], and Tamil Nadu [28]. Fungal keratitis is more frequently reported in winter with a humid climate favoring fungal growth [29, 30].

The generally accepted clinical features for the diagnosis of mycotic keratitis are the presence of a dry, raised ulcer with a feathery or hyphate border, satellite lesions, and recurrent hypopyon [31]. Our results are similar to reports from Delhi [2] and Madurai [30, 32]. The pigmented plaque like the presentation seen in 42% of our cases in the Dematiaceous group is similar to the series reported by Garg and associates in 2000 and 2004, respectively [33, 34].

Direct microscopy is an important diagnostic modality in investigating microbial keratitis, and a highest sensitivity at 99% is reported [35]. The addition of calcofluor white (CFW) stain to the diagnostic armamentarium has significantly increased the sensitivity of smear examination on direct microscopy [19]. However, it is difficult to determine the genus of fungi from KOH mounts [35]. Preliminary identification of *Fusarium* and *Aspergillus* species using microscopic features in histological specimens has been reported [36]. Identification of *Fusarium* from scraping material is reported by the detection of adventitious sporulation [37]. The presence of a brown colored fungal hyphae in the scraping material raised the possibility of the presence of a Dematiaceous mold [33]. Morphologically, we did not find any remarkable differences between the *Fusarium* and *Aspergillus* groups except that the distance between the septa was larger in *Fusarium* specimens. We established that hyphal thickness was greater in the Dematiaceous samples as compared to the specimens from other groups. Since brown coloration of the fungal cell wall may not be seen in all Dematiaceous cases [33, 38, 39], we believe that hyphal thickness can be used as an indication of Dematiaceous fungi.

Molecular identification of keratitis causing *Fusarium *and *Aspergillus* isolates has been reported earlier [9–12]. However, in cases of *Fusarium* and *Aspergillus*, it was established that the ITS region alone cannot discriminate between close species [40]. A comparative sequence analysis of other genes such as EF-1 and RPB2 for *Fusarium* and *β*-tubulin for *Aspergillus* is necessary for species identification within the complex. There is only one such study from India, where complete identification of *Aspergillus *species was done [10]. In a recent study on *Fusarium* keratitis where the ITS region was used, most isolates belonged to the *Fusarium solani* species complex [12]. In case of *Fusarium*, the MLST web site was found to be more convenient than NCBI. We found that a sequence comparison of the sole ITS region at the NCBI database accurately identified the Dematiaceous isolates.

Till date, four studies have explored the *in vitro* antifungal susceptibility patterns among keratitis-causing fungal isolates from India [13–16]. Our results are similar to those of Lalitha et al. [14] and show that amphotericin B and natamycin have the lowest MICs for *Fusarium* and *Aspergillus* species. A study on *Fusarium* keratitis isolates demonstrated high levels of *in vitro* resistance to voriconazole, amphotericin B, and natamycin [12]. Our results corroborate with their findings that, across the *Fusarium solani* species complex (FSSC), amphotericin B had the lowest MIC values. Antifungal susceptibility of *Aspergillus* species was recently reported in a study from India, and our MIC data is similar in the case of Fluconazole but slightly lower for Natamycin and Amphotericin B [10]. Studies on *Aspergillus* isolates from other infections also report low MIC ranges similar to ours for *A. fumigatus*, *A. flavus*, and *A. niger *[41]. In our study, only one isolate, *A. versicolor,* showed a high MIC value (16 *μ*g/mL) and these results are contradictory to the MIC values (1-2 *μ*g/mL) reported previously [42]. Antifungal susceptibility to *A. terreus* has been shown in many studies with a very high variability in MIC values [20]. In a report on keratitis causing *A. terreus *isolates, ketoconazole was shown to be the most effective agent [43]. Our results also show ketoconazole to be the most effective agent against *A. terreus*.

We found a good response in the pigmented keratitis group, and this was correlated with the lower MIC values. However, due to the small sample size, the results were not statistically significant. A similar observation was made earlier, where *in vitro* susceptibility to natamycin correlated with a favourable clinical response [16]. They attributed the good treatment outcome in pigmented keratitis to the low virulence of Dematiaceous fungi and their tendency to remain in the superficial tissues of the cornea. In another study, eyes with pigmented keratitis and nonpigmented keratitis showed hardly any difference in the medical response and visual outcomes [44]. Recently, in a study from south India, a significant association was made between corneal perforation and higher MIC values [13].

Despite the fact that we used a small sample size in each group, we noted pivotal dissimilarities between the groups. This study demonstrates important differences in microscopic features between *Fusarium*, *Aspergillus*, and *Dematiaceous* molds from clinical samples. The antifungal susceptibility results suggest that accurate identification would aid in specific treatment strategies.

## Figures and Tables

**Table 1:** Epidemiologic characteristics, risk factors, and clinical features.

Variable	*Fusarium* spp. (*n* = 26)	*Aspergillus* spp. (*n* = 15)	Dematiaceous isolates (*n* = 12)
Age (in years)	41.84	52.46	46.5
Gender ratio (male : female)	2.71	2	1.4
Risk factors			
Trauma other than vegetative body	1 (3.8%)	4 (26.6%)	0
Trauma with vegetative body	11 (42.3%)*	0	4 (33.3%)
Prior ocular surgery/infection	1 (3.8%)	5 (33.3%)	1 (8.3%)
Seasonal distribution			
Summer	6 (23%)	3 (20%)	1 (8.3%)
Monson	4 (15.3%)	7 (46.6%)	3 (25%)
Winter	14 (53.8%)	7 (46.6%)	6 (50%)
Clinical features			
Central location	23 (88.4%)	12 (80%)	11 (91.6%)
Size (>10 mm)	16 (61.5%)	9 (60%)	11 (91.6%)
Hypopyon	10 (38.4%)	5 (33.3%)	9 (75%)*
Elevated slough	19 (73.0%)	6 (40%)	10 (83.3%)
Dry texture	20 (76.9%)	7 (46.6%)	9 (75%)
Serrated ulcer margins	21 (80.7%)	9 (60%)	8 (66.6%)
Pigmentation	0	1 (6.6%)	5 (41.6%)
Endothelial plaque	4 (15.3%)	0	0
Dendritic lesions	0	2 (13.3%)	0
Satellite lesions	15 (57.6%)	8 (53.3%)	3 (25%)
Mean number of days for complete healing	57.4*	114.8	125.6

*Group significantly higher compared to other.

**Table 2:** *In vitro* susceptibility of *Fusarium*, *Aspergillus*, and Dematiaceous isolates to antifungal agents.

Isolate (number)	Agent, G-MIC* (range)			
	Natamycin	Amphotericin B	Fluconazole	Itraconazole
*Fusarium solani *(*n* = 22)	29.3 (8–128)	6.3 (1–32)	128 (≥128)	113.7 (64–128)
*Fusarium delphinoides *(*n* = 4)	12 (8–16)	12 (8–16)	128 (≥128)	128 (≥128)
*Aspergillus flavus *(*n* = 6)	32 (32)	0.35 (0.2–0.5)	128 (≥128)	1.5 (1–2)
*Aspergillus terreus *(*n* = 3)	13.3 (8–16)	2.5 (1–3)	26.6 (16–32)	0.25 (0.25)
*Aspergillus niger *(*n* = 1)	8	0.06	128	64
*Aspergillus tamarii *(*n* = 1)	64	0.25	≥128	0.25
*Aspergillus tubingensis *(*n* = 1)	8	2	32	0.25
*Aspergillus sydowii *(*n* = 1)	4	4	≥128	≥128
*Aspergillus versicolor *(*n* = 1)	4	2	32	0.25
*Curvularia lunata *(*n* = 4)	12.5 (2–32)	16 (0.25–32)	128 (≥128)	128 (≥128)
*Exherohelum rostratum *(*n* = 2)	2 (2–4)	1 (0.5–2)	32 (32)	0.25 (0.25–0.5)
*Cladorrhinum bulbilossum *(*n* = 1)	0.064	0.064	0.32	0.016
*Lasiodiplodia theobromae *(*n* = 1)	0.064	0.064	0.32	0.016
*Papulaspora* spp. (*n* = 1)	0.064	0.064	0.32	0.016

*Geometric mean of MIC.

**Table 3:** Clinical outcomes of *Fusarium*, *Aspergillus*, and Dematiaceous keratitis.

Groups	Mean visual acuity before treatment (LOGMAR)	Mean visual acuity after treatment (LOGMAR)	Change in visual acuity			Worsened
			Poor response (No PL to PL/PL to PR)	Slight response (improved Snelens/ CF to Snelens)	Good response (PL to CF/Snelens)	
*Fusarium *	1.82 (0.8–3)	1.17 (0–3)	3 (20%)	8 (53.3%)	3 (20%)	1 (6.7%)
*Aspergillus *	2.08 (0.48–3)	1.68 (0–3)	4 (16.7%)	12 (49.9%)	5 (20.9%)	3 (12.5%)
Dematiaceous	2.45 (0.48–3)	0.91 (0–2)	0	3 (30%)	7 (70%)*	0

*P value <0.05 when compared to the other two groups.
